# Agroecosystem vulnerability and driving factors in Northeast China

**DOI:** 10.1371/journal.pone.0339870

**Published:** 2026-02-12

**Authors:** Yu Jiang, Yitong Wang, Wenxin Zheng, Yufei Wang

**Affiliations:** College of Economics and Management, Northeast Forestry University, Harbin, Heilongjiang, China; Gebze Teknik Universitesi, TÜRKIYE

## Abstract

To realize the sustainable development of agro-ecology in Northeast China and help our country achieve the goal of wide-area agro-ecological maintenance, this study takes “sensitivity⁃resilience⁃pressure” as the evaluation model, selects 13 evaluation indicators and adopts the principal component analysis method to calculate and grade the vulnerability of agroecosystems in the three northeastern provinces and 36 prefectural-level cities from 2004 to 2022 using principal component analysis, and the driving factors were evaluated using parameter-optimized geodetector methods aiming at exploring the changes in spatial and temporal patterns of agroecological vulnerability in the northeastern region and the influence of the driving factors of each of its indicators on its vulnerability index. The conclusions are as follows: (1) In terms of temporal evolution, the overall vulnerability of agricultural ecosystems in Northeast China has shown a fluctuating downward trend. The proportion of different vulnerability levels has fluctuated significantly, with moderately vulnerable areas continuously transitioning toward mild vulnerability, indicating a positive trend. (2) In terms of spatial evolution, the vulnerability of the agricultural ecosystem in the northeastern region shows a distribution pattern of “high in the southwest, low in the northeast,” gradually decreasing from south to north, with an overall trend of gradual improvement. (3) In terms of driving forces, the spatial pattern is mainly determined by the terrain, with surface vegetation playing a core regulatory role by buffering climate pressures and enhancing ecological functions. At the same time, agricultural production conditions serve as a key human intervention to enhance resilience. This relies on the deep interaction between surface vegetation and human activity factors, which, through positive feedback between ecological and economic systems, and synergy between vegetation and irrigation, jointly drive the system’s resilience pattern.

## 1. Introduction

In recent years, due to the combined effects of human activity and global climate change, ecosystem vulnerability has emerged as a crucial topic for regional sustainable development studies. The Northeast region’s agro-ecosystem’s stability and health are much more crucial for the nation’s ecological balance and food security because it is a key barrier to ecological security and a region for food production in northern China. In light of this, China has developed the red line for the conservation of arable land, implemented policies for agricultural support and protection subsidies, and enhanced and fortified the agro-ecological security framework [1]. It is urgent to accurately recognize and construct a regional agroecological system vulnerability evaluation structure, examine its spatial and temporal evolution and influencing factors, and promote wide-area agroecological maintenance and achieve the goal of sustainable development.

Ecosystem vulnerability refers to the nature of various ecosystems that are easily damaged and difficult to recover to their original state under the influence of natural and anthropogenic factors [2,3]. In recent years, to explore ecosystem vulnerability in depth, scholars at home and abroad have adopted a variety of indicator evaluation models, including the sensitivity-resilience-pressure degree (SRP) model [4,5], the pressure-state-response (PSR) model [6,7], and the driving force-pressure-state-influence-response (DPSIR) model [8–10], to reveal the complex interactions between human activities and ecosystems in depth. And ecosystems. In addition, a variety of statistical analysis methods were utilized, such as the spatial distribution of ecological vulnerability by using the spatial principal component analysis (PCA) function [11–15], data analysis by adding seasonal trends, interaction factor analysis by using geoprobes [16,17], and calculation of the indicator bias, factor contribution, and barrier by using the barrier model [18,19], to explore the relationship between ecological vulnerability and various kinds of ecosystems. Calculations to explore the relationship between ecological vulnerability and various drivers [20,21]. In order to deal with uncertainty and ambiguity in the evaluation process, scholars have also applied comprehensive evaluation models [22,23], including the fuzzy comprehensive evaluation method [24] and hierarchical analysis method (AHP) [25,26], which are capable of both qualitative and quantitative analysis. Through the combined use of these interdisciplinary research methods and models, the aim is to provide a scientific and comprehensive evaluation model of ecosystem vulnerability and to provide a solid scientific basis for the sustainable development strategy of the region.

As China’s greatest agricultural commodities food base, the northeast area is crucial to maintaining the country’s economic growth. But in the Northeast, black soil degradation, forest area loss, and wetland function deterioration [27,28] have gotten worse as a result of fast industrialization, intensified agriculture, and urbanization. In addition, human activities, environmental pollution, and climate change have further aggravated the deterioration of agroecological vulnerability [29–31]. Therefore, innovation in technology and integrated management approaches is desperately needed to enhance soil quality, evaluate ecological resilience, and alleviate the contradiction between agriculture and ecological protection through a differentiated ecological compensation mechanism [32] to support China’s northeastern region’s sustainable development [33].

In summary, this study focuses on the Northeast region as its research subject, drawing on methods proposed by domestic and international scholars for establishing vulnerability assessment indicator systems [19,34,35], selecting frequently used indicators to construct an agricultural ecosystem vulnerability assessment model based on the “Sensitivity-Resilience-Pressure” (SRP) framework. Based on a comprehensive consideration of natural factors such as topography, climate conditions, and vegetation cover, as well as socio-economic factors like population density and economic growth, agricultural-related data were incorporated to select 13 key evaluation indicators [36]. Principal component analysis was used to calculate the comprehensive evaluation index of agricultural ecological vulnerability in Northeast China from 2004 to 2022, and the Optimal Parameters-based Geographical Detector (OPGD) was employed to evaluate the driving forces. The aim was to deeply analyze the spatiotemporal dynamic changes, vulnerability level distribution, and the impact of various driving factors on the agricultural ecological vulnerability index in Northeast China, to reveal the key influencing factors of agricultural ecological vulnerability in the region and to inform regional ecological environment risk management.

## 2. Materials and methods

### 2.1. Study area

The Northeast Region is situated in the center of Northeast Asia and includes the provinces of Heilongjiang, Jilin, and Liaoning. It is primarily situated between 42.3°N and 47.6°N and 119.1°E and 126.3°E. With a total area of roughly 7.873 x 105 km^2^, the region is primarily made up of plains, basins, hilly terrain, and mountains. In general, the geography of the Northeast Region is characterized by high altitude in the surrounding regions and low altitude in the core. With four distinct seasons and simultaneous heat and rain, it is located in the cold temperate and temperate monsoon climate zones, which provide perfect growing conditions for a variety of flora kinds. Corn, rice, soybeans, and sorghum are among the crops grown in this annual crop ripening system [37], which features a wide range of agroecosystem types.

Nevertheless, the issue of vulnerability in this region still warrants attention. In recent years, global climate change has had a significant impact on the ecosystems of Northeast China, disrupting vegetation growth and ecological balance. Soil erosion is severe in hilly and mountainous areas, leading to ecosystem degradation and weakened ecological functions, which in turn affect the sustainable development of agriculture. Therefore, studying the vulnerability of agricultural ecosystems in Northeast China can help us understand the stability and resilience of ecosystems in this region and provide support for regional sustainable development.

### 2.2. Framework for analyzing agro-ecosystem vulnerability mechanisms

Ever since the notion of “ecological vulnerability” was put forth, researchers have examined the mechanisms behind “agroecological vulnerability” from many angles. The scientific validity of evaluation indicators and the course of future development are directly tied to the definition of mechanism research. Based on previous theories of sustainable agricultural development [38,39], the economics of resilience theory, and China’s government policies [40–42], the mechanism of agroecological vulnerability was defined.

**Fig 1:**
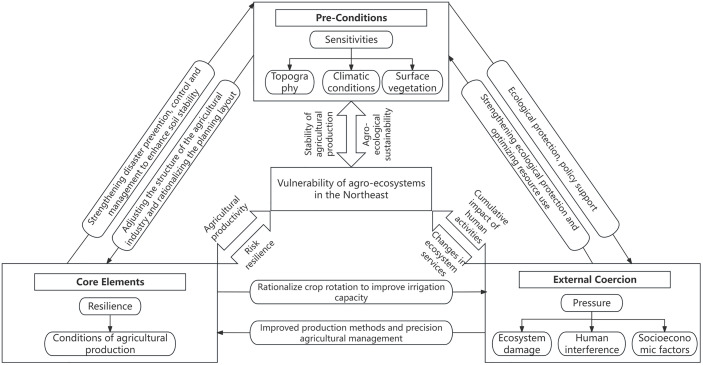
Mechanism analysis framework.

As can be seen from Fig 1, there is a cross-logical relationship between the three criterion layers of the SRP influencing factor evaluation system, and each criterion layer is further refined into several target layers, which have a clear scientific positioning in terms of spatial scale and mechanism of action, and assume specific functional modules, and systematically influence agro-ecological vulnerability through synergistic effects respectively. Specifically as follows:

Sensitivity analysis serves as a prerequisite for assessing the vulnerability of agricultural ecosystems in the Northeast region, revealing the sensitivity of agricultural ecosystems to natural and human-induced disturbances. Sensitivity indicators for topography, climate conditions, and surface vegetation characterize the vulnerability of agricultural ecosystems under environmental gradient changes. By adjusting agricultural industrial structures and rationally planning layouts, the intensity of the system’s response to natural disturbances can be reduced. Additionally, ecological protection policies (such as black soil protection policies, improving soil structure, enhancing soil erosion resistance, and reducing sensitivity risks exacerbated by soil degradation) resist ecological pressures caused by human activities, and thereby influence the stability and sustainability of agricultural production [43–45].

Resilience evaluation is a core element of vulnerability assessment for agricultural ecosystems in the Northeast region, representing the ability of agricultural ecosystems to recover to their initial state after disturbances. Resilience indicators for agricultural production conditions [46] are directly linked to the efficiency of agricultural production and its ability to withstand risks. On one hand, they strengthen disaster prevention and control, enhance soil stability, weaken the sensitive impacts of natural elements, and reduce the risk of ecosystems falling into a vulnerable state due to environmental fluctuations. On the other hand, they optimize agricultural production conditions through reasonable crop rotation, improved irrigation capacity, and other measures to alleviate the pressures caused by ecosystem destruction and human interference. Agricultural ecosystems with high resilience are more effective in responding to challenges posed by natural disasters and market fluctuations, thereby achieving sustainable development.

Stress analysis reveals the external pressures faced by agricultural ecosystems in the Northeast region, encompassing ecosystem degradation, human activity interference, and socioeconomic factors. It maps the cumulative impact of human activities on agricultural ecosystems and, through optimizing resource utilization and improving production methods, exerts a counteracting effect on sensitivity and resilience, forming a dynamic closed-loop system. Excessive human intervention and resource consumption may lead to the degradation of ecosystem services, thereby increasing the vulnerability of agricultural ecosystems [47–49].

The synergistic development of the aforementioned three criteria levels focuses on the long-term balanced connection between agricultural ecology, economy, and society, emphasizing rational resource utilization and ecological protection. This promotes industrial structure optimization and efficient resource allocation, further driving agricultural production in the Northeast toward sustainable development within the “ecological stability-productive efficiency-socioeconomic coordination” framework. It achieves a resilient cycle of “disturbance-response-balance” in agricultural ecosystems, effectively reducing their vulnerability.

### 2.3. Research methodology

#### 2.3.1. Vulnerability estimation method.

1. Evaluation indicators

Using the three characteristics of sensitivity, resilience, and stress, SRP is a model that may fully evaluate a region’s ecological vulnerability to the combined effects of anthropogenic and natural variables.

From the perspective of sensitivity, topographical factors, as natural baseline conditions, not only shape the spatial patterns and fundamental environment of agricultural ecosystems but also serve as the core foundational indicators for sensitivity assessment. Climate conditions and surface vegetation, as key drivers of dynamic changes in agricultural ecosystems, directly influence the survival status of flora and fauna. From the perspective of resilience, agricultural production conditions serve as the core foundation for ecosystems to achieve self-repair after disturbances—when production conditions are favorable, the system can rapidly restore ecological and production balance after being disrupted. In terms of stress intensity, ecosystem destruction directly alters ecosystem structure; human interference encroaches on agricultural ecological space; and socioeconomic factors indirectly influence the stability of agricultural ecosystems through policy direction and resource allocation.

It is important to note that Northeast China’s industrialization and urbanization processes have been steadily progressing in recent years. This study specifically introduces nighttime light data as a quantitative indicator of human disturbance intensity in order to more accurately measure the pressure of human activities (e.g., urban expansion encroaching on farmland) on agricultural ecosystems at the macro level and more intuitively reflect the intensity and scope of human activities.

In summary, this study follows the principles of comprehensiveness, scientificity, comparability, hierarchy, stability and data availability, refers to the relevant vulnerability evaluation index system and research results in the field of agriculture [19,34,35], selects the representative indicators that have been widely used in domestic and international research, combines the indicator layer selection specification of the SRP model and the characteristics of agro-ecosystems of Northeast China, and ultimately determines 13 indicators to build the The ecological vulnerability evaluation index system of the Northeast region, and through the positive and negative indicators to clarify the direction of the influence of each indicator on ecological vulnerability [50], the specific indicators are shown in Table 1.

**Table 1:** Evaluation indicator system.

Target Level	Standardized Layer	State Layer	Indicator Stratum (units)	Causality
Vulnerability of agro-ecosystems in the Northeast	Sensitivities	Topography	Altitude (m)	+
			Elevation (m)	+
		Climatic conditions	Dryness index (-)	+
			Average annual precipitation (mm)	–
			Average annual temperature (degrees)	+
		Surface vegetation	Normalized vegetation factor (-)	–
			Vegetation cover (%)	–
	Resilience	Conditions of agricultural production	Agricultural power per unit area (kw/ha)	–
			Irrigation index (%)	–
	Stress level	Ecosystem damage	Agricultural fertilizer use per unit area (t/ha)	+
		Human interference	Nighttime Lighting Data (-)	+
		Socio-economic factors	GDP per capita (￥)	+
			Population density (persons/km2)	+

Among them, positive indicators show that ecosystems are more susceptible to external perturbations, while negative indicators show that ecosystems are less susceptible.

2. Standardization of indicators

The data were standardized in this study using the method of standardization of extreme deviation [41–52]. By combining positive and negative indicators, the loadings of principal components can be interpreted more intuitively as the direction of the contribution of the original indicators, enhancing the interpretability of the principal component loadings.

Positive indicators:


$$X_{ij}=\frac{x_{ij}-x_{min}}{x_{max}-x_{min}}$$ (1)

Reverse indicators:


$$X_{ij}=\frac{x_{max}-x_{ij}}{x_{max}-x_{min}}$$ (2)

Where $x_{ij}$ is the original data, $X_{ij}$ is the normalized data, and $x_{max},x_{min}$ are the corresponding maximum and minimum values.

3. Principal component analysis

13 evaluation indicator elements of agro-ecological vulnerability in the three northeastern provinces and 36 prefectural-level cities were subjected to principal component analysis (PCA) in this study, which was based on the principal component analysis function of SPSS [50,51]. Multiple evaluation indicators were downscaled into a number of comprehensive and unrelated factors by rotating the original variables’ axes. This reduced the amount of data while preserving the great majority of the original variables’ information.

The formula is as follows:


$$\omega_{i}=\frac{\lambda_{i}}{\sum_{i=1}^{n}\lambda_{i}}$$ (3)

Where $\omega_{i}$ is the corresponding contribution rate of the *ith* principal component, and $\lambda_{i}$ is the eigenvalue of *the ith* principal component.

On this basis, the first five principal components with a cumulative variance contribution of 80% or more were selected to replace the original indicator factors to derive the agro-ecological vulnerability index [18,52–54] for each region in each calendar year with the following formula:


$$EVI=\sum_{i=1}^{n}\omega_{i}\times PC_{i}$$ (4)

Where EVI is the vulnerability index of agroecosystems in Northeast China; $\omega_{i}$ is the ith primary component’s matching contribution rate; $PC_{i}$ is the ith principal component.

Therefore, the agro-ecological vulnerability index was constructed for each year as follows:


$$EVI_{2004}=0.333PC_{\text{1}}+0.159PC_{\text{2}}+0.147PC_{\text{3}}+0.091PC_{\text{4}}+0.074PC_{\text{5}}$$ (5)

$$EVI_{2007}=0.229PC_{\text{1}}+0.215PC_{\text{2}}+0.167PC_{\text{3}}+0.128PC_{\text{4}}+0.079PC_{\text{5}}$$ (6)

$$EVI_{2010}=0.350PC_{\text{1}}+0.196PC_{\text{2}}+0.165PC_{\text{3}}+0.075PC_{\text{4}}+0.071PC_{\text{5}}$$ (7)

$$EVI_{2013}=0.296PC_{\text{1}}+0.202PC_{\text{2}}+0.165PC_{\text{3}}+0.096PC_{\text{4}}+0.072PC_{\text{5}}$$ (8)

$$EVI_{2016}=0.328PC_{\text{1}}+0.179PC_{\text{2}}+0.152PC_{\text{3}}+0.095PC_{\text{4}}+0.064PC_{\text{5}}$$ (9)

$$EVI_{2019}=0.378PC_{\text{1}}+0.162PC_{\text{2}}+0.124PC_{\text{3}}+0.093PC_{\text{4}}+0.066PC_{\text{5}}$$ (10)

$$EVI_{2022}=0.312PC_{\text{1}}+0.153PC_{\text{2}}+0.147PC_{\text{3}}+0.090PC_{\text{4}}+0.073PC_{\text{5}}$$ (11)

4. Agro-ecological vulnerability classification

With the goal of more intuitively investigating the extent of regional differentiation of agroecosystem vulnerability in the three northeastern provinces and prefecture-level cities, *the EVI* index was graded and categorized. Before that, it was standardized [55]:


$$S_{EVI}=\frac{EVI-EVI_{min}}{EVI_{max}-EVI_{min}}$$ (12)

Where, $S_{EVI}$ is the Northeast region’s standardized agro-ecological vulnerability index score, which goes from 0 to 1; $EVI_{max}$ is the Northeast region’s highest score on the agro-ecological vulnerability index; $EVI_{min}$ is the Northeast region’s agro-ecological vulnerability index’s lowest value.

Based on the existing domestic and international studies [56,57], the agroecological vulnerability of the Northeast region was further divided into five classes, and the SRP model was analyzed to correspond to the agroecological characteristics and the specific division criteria are listed in Table 2:

**Table 2:** Vulnerability classification.

Level of vulnerability	Standardized values for agricultural vulnerability indicators	Vulnerability	Agro-ecological characteristics
Level 1	0 ≤ *SEVI* < 0.2	Slightly vulnerable	The agro-ecological system in the Northeast has a perfect structure and function, strong resistance to external disturbances, low stress, strong resilience, stable agro-ecosystems, and low agro-ecological vulnerability.
Level 2	0.2 ≤ *SEVI* < 0.4	Mildly vulnerable	The agroecosystems of the Northeast are better structured and functional, more resistant to external disturbances, less stressful, more resilient, with more stable agroecosystems and lower agroecological vulnerability.
Level 3	0.4 ≤ *SEVI* < 0.6	Moderately vulnerable	The structural functions of agroecosystems in the Northeast region can still be maintained, with weak resistance to external disturbances, near-threshold pressure, weak resilience, unstable agroecosystems, and high agroecological vulnerability.
Level 4	0.6 ≤ *SEVI* < 0.8	Highly vulnerable	The structural and functional defects of agroecosystems in the Northeast include weak resistance to external disturbances, high pressure, unstable agroecosystems, more difficult recovery after damage, and high agroecological vulnerability.
Level 5	0.8 ≤ *SEVI*≤ 1	Extreme vulnerability	The structure and function of the agro-ecological system in the northeast region are not perfect, the ability to resist external interference is extremely weak, the pressure to bear is extremely great, the agro-ecological system is extremely unstable, and the difficulty of recovering from damage is extremely great, and it may even be difficult to reverse, so the agro-ecological vulnerability is extremely high.

#### 2.3.2. Geoprobes based on optimal parameters.

Scholar Wang Jinfeng [58] proposed the geographic detector as a statistical model to identify spatial differentiation and uncover its underlying causes. The variation of the three northeastern provinces’ spatial distribution layer observation values and the driving forces is represented by the q-statistic [59,60]. The greater the q-statistic, the more effective the discretization classification is. It can be used to evaluate the efficacy of discretization classification. The discretization settings for traditional geographic detectors must be manually set, which is quite subjective and frequently leads to subpar discretization.

We develop an optimal parameter-based geodetector using equal breaks, natural breaks, quantile breaks, geometric breaks, and standard deviation breaks with the help of the GD package in the R language [61–63]. Two to eight levels of categorization were established using equal breaks, natural breaks, quantile breaks, geometric breaks, and standard deviation breaks. The parameter for the study of the geodetectors was determined to be the spatial scale with the highest q-value.

(1) Divergence and Factor Detector

To determine the spatial heterogeneity of the dependent variable EVI (agro-ecological vulnerability) and the extent to which factor X explains the spatial heterogeneity of EVI, the formula was calculated as:


$$q=1-\frac{\sum_{h=1}^{L}N_{h}\sigma_{h}^{\text{2}}}{N\sigma^{\text{2}}}=1-\frac{SSW}{SST}$$ (13)

$$SSW=\sum_{h=1}^{L}N_{h}\sigma_{h}^{\text{2}},SST=N\sigma^{\text{2}}$$ (14)

Measuring the degree to which the driving factor X explains the spatial differentiation of the dependent variable EVI is the fundamental purpose of the q statistic. The study area is separated into multiple layers (categories) according to the values of factor X, and the sum of the variances of EVI within each layer (SSW) is computed about the total variance of EVI throughout the entire area (SST). This computation is based on the principles of analysis of variance, and [0,1] is the range of values. A q value near 0 suggests that factor X has little explanatory power for the spatial differentiation of EVI, whereas a q value near 1 better explains the geographical distribution pattern of EVI (high variations between layers and modest changes within layers). h = 1, 2..., L is the stratification of EVI or X; Nh, N is the number of cells in stratum h and the entire area, respectively; σ2h, σ2 is the variance of EVI values in stratum h and the entire area, respectively. The intra-layer variation and the overall variance for the entire region, respectively; SSW and SST represent the variance of the EVI values for the entire region.

(2) Interaction detectors

Determine how various factors Xs interact with one another by comparing the explanatory power of the dependent variable EVI when factors X1 and X2 work in tandem versus when they act independently. First, the q-values of the two factors X1 and X2 on EVI are calculated individually (q(X1) and q(X2)), and then the q-value of the new polygon distribution created by their interaction (q(X1∩X2)) is calculated. Finally, q(X1), q(X2), and q(X1∩X2) are compared. Table 3 lists the association between the two factors in terms of classification.

**Table 3:** Classification of the relationship between the two factors.

Geographical interaction relationship	Interaction	Potential Causal Mechanism
q(X1∩X2)<min[q(X1),q(X2)]	Nonlinear-weakened	There are buffering or inhibitory effects between factors. For example, factor A may offset or mitigate the negative impact of factor B, resulting in a total impact that is less than the minimum impact of either factor acting alone when the two coexist.
min[q(X1),q(X2)]≤q(X1∩X2)≤max[q(X1),q(X2)]	Uni-variables weaken	The interaction did not enhance the dominant factor or slightly weakened it. The synergistic effect was mainly determined by the more influential factor, and the addition of the other factor did not significantly enhance the effect, but even slightly weakened the influence of the dominant factor.
max[q(X1),q(X2)] <q(X1∩X2) < q(X1)+q(X2)	Bi-variables enhance	There is a synergistic amplification effect between factors (but not extreme ones). When two factors act together, they promote or amplify each other’s effects, resulting in a total impact greater than the strongest single factor, but not yet reaching the level of simple addition.
q(X1∩X2) =q(X1)+q(X2)	Independent	The effects of the factors are independent of each other. The paths through which the two factors influence vulnerability do not interfere with each other, and their combined effect is equal to the linear sum of their individual effects.
q(X1∩X2) >q(X1)+q(X2)	Nonlinear-enhancement	There is a strong nonlinear amplification or threshold effect. When the two factors act together, they trigger a violent reaction that far exceeds the sum of their independent effects (such as ecological collapse and resource depletion thresholds).

### 2.4. Data sources

Table 4 of this study contains topographic data, meteorological data, vegetation data, agricultural data, and socioeconomic and demographic data for the three northeastern provinces and their 36 prefectural-level cities from 2004 to 2022, with some missing data estimated and replaced using linear interpolation methods.

**Table 4:** Data sources.

data type	data name	Data sources	time resolution	spatial resolution	particular year
Topographic data	Elevation DEM data	Data Center for Resource and Environmental Sciences, Chinese Academy of Sciences (http://www.resdc.cn)	–	30m	–
	Slope data		–	30m	–
Climate data	Dryness index		Year	–	
	Average annual precipitation	The raw data were obtained from the National Center for Environmental Information (NCEI) under the National Oceanic and Atmospheric Administration (NOAA) of the United States of America, and interpolated to obtain prefecture-level city data based on the station data and administrative boundary data using GIS inverse distance interpolation calculations.	Year	1km	2004-2022
		Average annual temperature	Statistical Yearbook of Provinces and Prefectural Municipalities	Year	–
Vegetation data	Normalized vegetation index NDVI	Derived from the MOD13A3 dataset regularly released by NASA, with official month-by-month NDVI data from February 2000 onwards (https://www.earthdata.nasa.gov/)	Month	500m	
		Vegetation cover	Data shared by Gao Jixi scholars on the National Tibetan Plateau Science Data Center platform. (https://data.tpdc.ac.cn/zh-hans/data/f3bae344-9d4b-4df6-82a0-81499c0f90f7)	Year	500m
Agricultural data	Agricultural power per unit area	Statistical Yearbook of Provinces and Prefectural Municipalities	Year	–	
		Irrigation index		Year	–
Socio-economic and demographic data	Agricultural fertilizer use per unit area (t/ha)	Statistical Yearbook of Provinces and Prefectural Municipalities	Year	–	
		Nighttime Lighting Data		Year	–
		GDP per capita ($)		Year	–
		Population density (persons/km2)		Year	–

## 3. Results

### 3.1. Characterizing changes in agro-ecosystem vulnerability

#### 3.1.1. Temporal evolution of vulnerability.

Combining the source data, the 13 evaluation factors in the agro-ecological vulnerability evaluation system of the Northeast region for the three years of 2004, 2013 and 2022 were subjected to principal component analysis, and the 13 principal component eigenvalues, contribution rates and cumulative contribution rates corresponding to each year were calculated and obtained, and the results of the calculations are shown in Table 5.

**Table 5:** Principal Component Analysis Results.

principal components	2004			2013			2022		
	characteristic value	Contribution rate	Cumulative contribution rate	characteristic value	Contribution rate	Cumulative contribution rate	characteristic value	Contribution rate	Cumulative contribution rate
1	4.335	33.345	33.345	3.844	29.567	29.567	4.056	31.202	31.202
2	2.067	15.897	49.241	2.626	20.2	49.767	2.477	19.056	50.258
3	1.906	14.658	63.899	2.149	16.534	66.301	1.983	15.255	65.512
4	1.185	9.117	73.016	1.25	9.616	75.917	1.175	9.042	74.554
5	0.963	7.404	80.421	0.932	7.171	83.089	0.947	7.283	81.837
6	0.781	6.006	86.426	0.666	5.124	88.213	0.819	6.299	88.136
7	0.706	5.433	91.859	0.646	4.97	93.183	0.533	4.103	92.239
8	0.301	2.314	94.173	0.305	2.342	95.525	0.412	3.169	95.408
9	0.296	2.276	96.45	0.208	1.598	97.123	0.255	1.963	97.371
10	0.239	1.84	98.289	0.15	1.151	98.274	0.178	1.366	98.737
11	0.101	0.778	99.067	0.108	0.828	99.102	0.076	0.582	99.319
12	0.082	0.631	99.698	0.066	0.51	99.612	0.055	0.421	99.74
13	0.039	0.302	100	0.05	0.388	100	0.034	0.26	100

**Fig 2:**
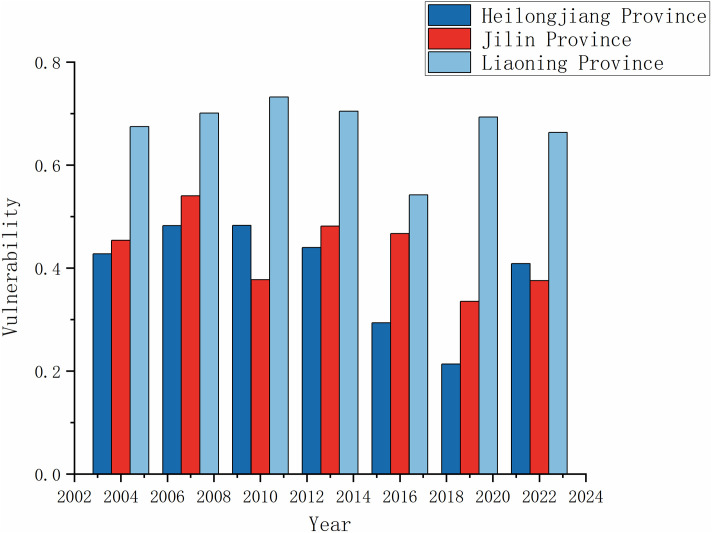
Temporal evolution analysis of agricultural ecosystem vulnerability in Northeast China.

Based on the calculation of the vulnerability index of agroecosystems in Northeast China from 2004 to 2022, the time-varying status of agroecosystem vulnerability in Northeast China from 2004 to 2022 was plotted, and the results are shown in Fig 2.

A comprehensive analysis shows that the vulnerability of agroecosystems in the northeast region declined from 0.52 in 2004 to 0.48 in 2022, with an overall fluctuating downward trend. Among them, the vulnerability of the agricultural ecosystem in Heilongjiang Province fluctuated from 0.43 in 2004 and decreased to 0.41 in 2022; the vulnerability of agroecosystems in Jilin and Liaoning Provinces is relatively stable, with Jilin Province slowly fluctuating from 0.45 in 2004 to 0.38 in 2022; In Liaoning Province, the vulnerability index fluctuates from 0.67 in 2004 to 0.66 in 2022.

From the perspective of the proportion of ecological vulnerability levels across cities in various provinces over different years, the overall ecological vulnerability of the Northeast region remained at a moderate level from 2004 to 2022. However, the proportions of different vulnerability levels fluctuated significantly. Moderately vulnerable areas continued to transition toward lightly vulnerable areas, driving an overall improvement in system stability. As a result, the ecological vulnerability of agriculture in the Northeast region showed a positive trend. Among these, the proportion of cities classified as slightly vulnerable increased significantly from 25.0% in 2004 to 41.7% in 2022, becoming the dominant vulnerability level; Meanwhile, the proportion of cities classified as severely vulnerable or higher decreased from a peak of 74.9% in 2013 to 52.8% in 2022, while the proportion of extremely vulnerable cities dropped from 22.2% in 2013 to 16.7% in 2022, a decrease of 24.8%. This reversal is primarily attributed to the “Outline of the Northeast Black Soil Protection Plan” (2017–2030) [64], which has effectively improved the regional ecological environment through systematic black soil protection and restoration measures. However, it is worth noting that two structural challenges remain: the proportion of moderately vulnerable cities has fluctuated without showing substantial growth, and in 2022, severely vulnerable cities and above still accounted for a high proportion of 30.6%. There is an urgent need to strengthen governance measures for water resource optimization in the arid belt of western Liaoning, such as Chaoyang and Fuxin cities, and for the remediation of contaminated sites in industrial and mining cities such as Anshan and Benxi cities.

#### 3.1.2. Spatial evolution of vulnerability.

**Fig 3:**
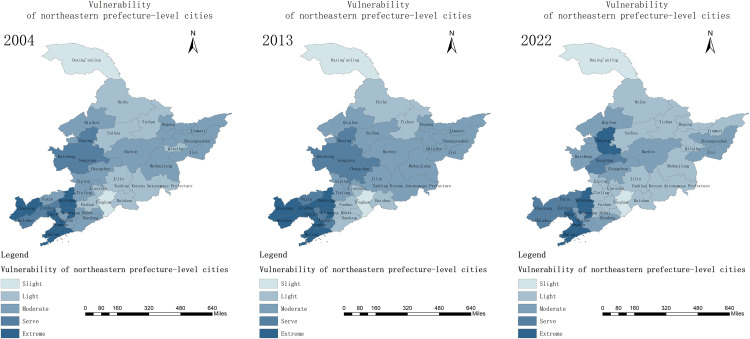
Spatial evolution analysis of agricultural ecosystem vulnerability in Northeast China.

Note: This map is produced according to the Chinese standard map with review number GS(2024) 0650 provided by the National Geographic Information Public Service Platform, with no modification to the base map.

By calculating the agroecosystem vulnerability data of each prefecture-level city in the Northeast region, ArcGIS 10.8 was used to draw the agroecosystem vulnerability zoning maps in 2004, 2013, and 2022, as shown in Fig 3.

In terms of spatial distribution, the vulnerability of agroecosystems in the northeast region from 2004 to 2022 shows a distribution pattern of “high in the southwest and low in the northeast”, with a gradual decrease from the south to the north and an overall trend of gradual improvement. This distribution pattern is mainly driven by the dual mechanism of natural conditions and human activities. The northeastern part of the country is more resistant to disturbance by virtue of its high precipitation rate, low risk of drought, and stable black soil base, while the southwestern part is less resistant to disturbance due to more serious soil erosion, strong dependence on chemical fertilizers, and over-cultivation with higher population density. The nature of this differentiation is the result of the combined effect of natural background differences and human regulatory capacity.

Among them, agro-ecological vulnerability showed a small upward fluctuation from 2004 to 2013, with the average EVI value fluctuating from 0.52 to 0.54: during this period, under the influence of the overuse of regional fertilizers, the mildly fragile zones in Suihua, Jilin, Yanbian Korean Autonomous Prefecture, and Qitaihe transformed into moderately fragile zones, and the agro-ecological environment carrying pressure intensified;Jinzhou, Huludao and other areas in western Liaoning Province, due to the double effect of the increase in dryness index and the increase in population density, the vulnerability of the increase is more obvious, from the severely vulnerable area to the extremely vulnerable area; Dandong, thanks to the good performance of indicators such as Normalized Vegetation Factor (NVF) and the local emphasis on ecological protection, the vulnerability index of its moderately vulnerable area was reduced from 0.53 to 0.32, showing a decreasing trend.

Thereafter, vulnerability shows a significant decreasing trend from 2013 to 2022, with the average EVI value decreasing from 0.54 to 0.48: with the drying index falling back and population density decreasing, the extremely vulnerable areas in Chaoyang and Huludao have shrunk; most of the moderately vulnerable areas, such as Jiamusi, Mudanjiang, and Siping City, have seen a reduction in ecological damage and have been transformed into mildly vulnerable areas, with the stability of agro-ecosystems enhanced; at the same time, Changchun City and Baicheng City and other places are slowly decreasing their vulnerability due to the increase in the irrigation index and the strengthening of agro-ecosystem resilience [36].

It is worth noting that despite a slight increase in regional ecological vulnerability between 2004 and 2013, during this period, in line with the long-term trend, moderate climate change, in conjunction with human intervention measures, continued to gradually build up regional ecological resilience: on the one hand, the aridity index in the three northeastern provinces continued to decline slowly (an average annual decrease of 3.15%), and vegetation coverage continued to show an upward trend (an average annual increase of 4.92%); on the other hand, the amount of fertilizer used per unit area has been decreasing annually (with an average annual decrease of 1.02%). This fully reflects the growing importance people place on ecological and environmental protection. Over the past two decades, the resilience of the agricultural ecosystems in the three northeastern provinces has continued to strengthen, with overall vulnerability levels showing a fluctuating downward trend.

### 3.2. Analysis of agroecosystem vulnerability driving mechanisms

#### 3.2.1. Optimal parameter selection.

The q-value for each continuous driver is determined by counting the number of categories that fall under several categorization techniques, including equal breaks, natural breaks, quantile breaks, geometric breaks, and standard deviation breaks), standard deviation, geometric breaks, and the q-value that corresponds to the number of distinct categories to eliminate the best scale for discretizing geographic data. Using 2022 as an example, Table 6 displays the final selection of the parameter combinations with the highest q-value.

**Table 6:** Parameter combinations with the largest values of the driving factor q.

driving factor	encodings	discretization method	Number of classifications
altitude (e.g., above street level)	X1	Geometric breaks	2
elevation	X2	Geometric breaks	8
dryness index	X3	Natural breaks	8
Average precipitation	X4	Geometric breaks	2
average temperatures	X5	Geometric breaks	2
Normalized vegetation factor	X6	Geometric breaks	4
vegetation cover	X7	Geometric breaks	5
Agricultural power per unit area	X8	Geometric breaks	2
irrigation index	X9	Geometric breaks	2
population density	X10	Geometric breaks	7
Average nighttime lighting data	X11	Geometric breaks	3
GDP per capita	X12	Geometric breaks	3
Fertilizer use per unit area	X13	Geometric breaks	2

#### 3.2.2. Driving factor analysis.

(1) Divergence and Factor Detector

**Fig 4:**
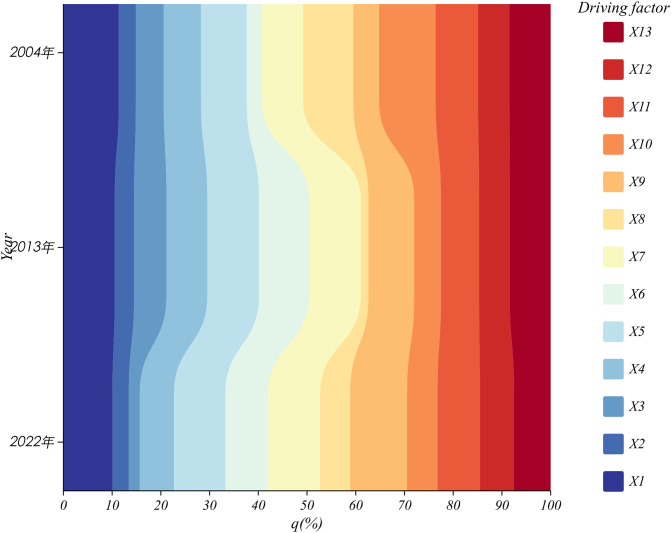
Plot of percentage explanatory power of driving factors over time.

According to the optimal parameter combinations to detect the explanatory power of the factors, Table 7 was made, and ChiPlot (https://www.chiplot.online/) was used to plot Fig 4. Each factor has obvious differentiation and evolution in the vulnerability of agroecosystems in the three northeastern provinces.

**Table 7:** Drivers 2004, 2013, 2022 q-values.

Driver encoding	2004		2013		2022	
	q-value	p-value	q-value	p-value	q-value	p-value
X1	0.870	0.000	0.795	0.000	0.822	0.000
X2	0.266	0.653	0.299	0.570	0.273	0.522
X3	0.438	0.013	0.502	0.017	0.184	0.871
X4	0.585	0.000	0.632	0.000	0.572	0.000
X5	0.715	0.000	0.800	0.000	0.861	0.000
X6	0.241	0.299	0.773	0.000	0.716	0.000
X7	0.644	0.000	0.802	0.000	0.868	0.000
X8	0.788	0.000	0.119	0.848	0.502	0.003
X9	0.404	0.096	0.708	0.000	0.953	0.000
X10	0.888	0.000	0.414	0.006	0.511	0.056
X11	0.666	0.001	0.592	0.011	0.709	0.000
X12	0.491	0.020	0.475	0.067	0.572	0.000
X13	0.646	0.000	0.632	0.005	0.612	0.000

In terms of heterogeneity, in 2004, X10 (fertilizer use per unit area), X1 (elevation), X8 (power of agricultural machinery per unit area), X5 (average air temperature), and X11 (average value of nighttime lighting data) were the main driving factors, with q-value sizes of 0.888, 0.870, 0.788, 0.715, and 0.666, respectively, which passed the test of significance. In 2013, X7 (vegetation cover), X5 (average temperature), X1 (elevation), X6 (normalized vegetation factor), and X9 (irrigation index) were the primary driving factors, with q-values of 0.802, 0.800, 0.795, 0.733, and 0.708, respectively, all of which passed the significance test; In 2022, X9 (irrigation index), X7 (vegetation cover), X5 (average temperature), X1 (elevation), and X6 (normalized vegetation factor) were the primary driving factors, with q-values of 0.953, 0.868, 0.861, 0.822, and 0.716, respectively, all of which passed the significance test. Overall, considering only significant factors, the average q-value for topography was the highest at 0.829, followed by surface vegetation and agricultural production conditions, with an average q-value of 0.741. These three factors play a dominant role in agricultural ecosystem vulnerability, reflecting the mutual influence between humans and nature.

In terms of evolvability, X1 (elevation), X5 (average temperature), X7 (vegetation cover) continued to maintain ultra-high explanatory power; X9 (irrigation index), X6 (normalized vegetation factor) generally showed an upward trend, highlighting the enhancement of the role of the improvement of agricultural infrastructure and ecological protection projects on the ecological regulation of farmland; X9 (irrigation index) increased the most, at 0.549, replacing the X3 (dryness index), which may become an important factor affecting ecosystem vulnerability in the future; X10 (fertilizer use per unit area) and X8 (agricultural machinery power per unit area) both fluctuated downward, down by 0.377 and 0.286 respectively, reflecting the differentiation between the promotion of the fertilizer reduction policy and the demand for large-scale cultivation, and between the popularization of agricultural machinery and the application of intelligent equipment, which may exit in the future. Phases, which may exit the dominant position in influencing the vulnerability of farmland ecosystems in the three eastern provinces in the future. The underlying reason for the persistent weakness of the X2 (slope) driver (three-year q-value <0.299) and the failure of the significance test (p > 0.522) is that the three northeastern provinces are dominated by the plains, and the overall gentle topography leads to the weak spatial differentiation of the slope, which makes it difficult to form an effective mechanism of differentiation of the vulnerability of the ecosystems. Overall, the Northeast farmland ecosystems have initially realized the transition of “pressure reduction - sensitivity weakening - resilience enhancement”, marking the evolution from high-input-dependent agriculture to a techno-ecological synergistic sustainable model.

(2) Interaction Factor Detector

**Fig 5:**
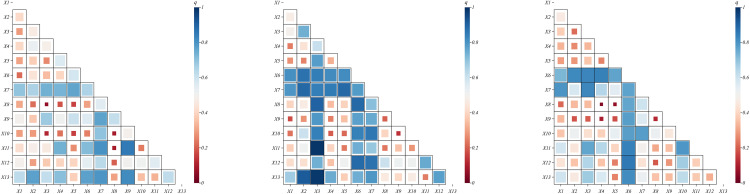
Heat map of factor interaction detection results (from left to right, 2004, 2013, 2022).

Meanwhile, 13 factors were detected for interaction, and correlation heatmaps were plotted using ChiPlot (https://www.chiplot.online/), and the results are shown in Fig 5.

From the perspective of the structural differentiation of the interaction types among the driving factors of agricultural ecosystem vulnerability, at the three time points of 2004, 2013, and 2022, the interactions between all factors and the vulnerability of farmland ecosystems in the three northeastern provinces are closely related. The driving factors do not act independently but exhibit a synergistic enhancement effect. This indicates that the interactions between any two of the 13 driving factors in this study have a more significant driving effect on vulnerability, meaning that the interactions between factors better explain regional differences in vulnerability than individual factors alone. Specifically, in 2004, the interaction between two factors dominated (accounting for approximately 65%), with the interaction between X9 (irrigation index) and X11 (nighttime light data) having the highest explanatory power (q = 0.855), reflecting the synergistic effects of agricultural water infrastructure (irrigation) and urbanization expansion (as indicated by nighttime light data). Irrigation projects rely on energy inputs, while urbanization intensifies agricultural electricity demand. The combination of these factors leads to water resource competition, amplifying ecological pressures. In 2013, the primary driver was the interaction of two factors (accounting for approximately 60%), but a nonlinear enhancement phenomenon emerged, with the strongest interaction between X3 (dryness index) and X13 (population density) (q = 0.986), revealing the overlapping effects of drought-prone areas and densely populated regions. The Songnen Plain and other arid regions are also major corn-producing areas and population-dense regions. Climate drought transmits pressure through grain production to the population-bearing system, forming a vulnerability amplification loop. In addition, the interaction between X8 (agricultural machinery power per unit area) and X3 (dryness index) also showed significant nonlinear enhancement (q = 0.902), indicating that in drought-prone areas, the investment in agricultural machinery power can significantly enhance the resilience of agricultural ecosystems through improving irrigation efficiency and farming practices, thereby mitigating the negative impacts of drought. In 2022, the dual-factor enhancement remained dominant (accounting for approximately 70%), with the highest explanatory power from the interaction between X6 (normalized vegetation factor) and X12 (per capita GDP) (q = 0.8582). High vegetation coverage areas (such as the Greater and Lesser Khingan Ranges) leverage ecological resources to develop green industries, while low coverage areas (the Liaoning Central-Southern Urban Agglomeration) implement ecological restoration through economic investments, forming a positive feedback loop.

From the temporal variation characteristics of the driving factors of farmland ecosystem vulnerability, the interactive driving mechanism of farmland ecosystem vulnerability exhibits a “synergistic deepening of natural and human factors” pattern: X11 (nighttime light data) and X3 (dryness index) are the primary interactive driving factor combination, with their interactive effect peaking in 2013 (q = 0.9891), Secondly, the interactive effects of X6 (normalized vegetation factor) and X9 (irrigation index), as well as X12 (per capita GDP) and X13 (population density), continued to strengthen (with q values exceeding 0.8 in 2022), indicating that the alignment between vegetation recovery capacity, agricultural investment levels, economic development, and population distribution is a key factor in the evolution of vulnerability. For the high-frequency interaction factors X11 (nighttime light data) and X6 (normalized vegetation factor), their interaction relationship is primarily driven by a two-factor enhancement (three-year q mean > 0.8), with efficiency improvements exceeding 40% compared to single-factor interactions. This suggests that, in addition to increasing vegetation coverage, regulating the intensity of human activities (such as optimizing urban expansion as indicated by nighttime light patterns) can effectively enhance system resilience. The study indicates that the sustainability of northeastern farmland ecosystems depends on the synergistic evolution of natural resilience and human regulatory capacity, necessitating the strengthening of system resilience through the integration of ecological conservation and agricultural intensification policies.

## 4. Discussions

As a way to demonstrate the scientific nature of this study, its marginal contribution, and its viability, it is compared to earlier research based on the aforementioned discoveries.

Overall, the Northeast region’s agricultural ecosystems’ sensitivity varies significantly over gradients. The vulnerability level of the agricultural ecosystems examined in this research in the Northeast region is generally consistent with China’s actual situation, as evidenced by this characteristic, which is consistent with previous studies and closely related to the overall vulnerability gradient differentiation of China’s agricultural ecosystems [65]. Simultaneously, by comparing various indices, such as the Gini index [66] and methods, such as the weighting method [67], for assessing the vulnerability of the three northeastern provinces, it is evident that the EVI index calculation’s results are consistent with those of other mainstream assessment methods in terms of classification and numerical trend, and the numerical deviation is within an acceptable range. This fully demonstrates the EVI index’s reasonableness and the dependability of its findings. From 2004 to 2022, agricultural drivers shifted from a high-input dependency model reliant on chemical fertilizers, pesticides, and traditional tillage to a sustainable, technology-ecology synergistic model supported by technologies such as smart irrigation and conservation tillage [28]. The impact of fertilizer use per unit area has dramatically decreased since the “Zero Growth in Fertilizer Use Action” and agricultural non-point source pollution control policies were put into place. This indicates that the trend of lowering ecological pressure from high-input agricultural models has entered a substantive phase. In the meantime, the ongoing improvement of irrigation indices and surface vegetation variables shows that ecological regulating capacity—rather than resource consumption—has emerged as the primary force behind boosting system resilience. This aligns closely with China’s ecological protection red line policy. This policy, through strict protection of key ecological spaces such as forests, grasslands, and wetlands (e.g., the Greater and Lesser Khingan Ranges, Changbai Mountains), has effectively maintained and enhanced regional vegetation coverage and ecosystem productivity, strengthened the system’s ability to buffer climate pressures (e.g., mitigating the negative impacts of the aridity index) and maintain ecological functions, making it a critical human intervention measure to reduce the vulnerability of the Northeast agricultural ecosystem. Elevation significantly influences water-heat conditions, suitability for cultivation, land reclamation and settlement patterns (e.g., land reclamation is primarily concentrated in plains), and its impact intensity ranks among the top five across all three periods. Additionally, the interactive effects of Northeast China’s agricultural ecosystem vulnerability reveal key controllable pathways, primarily involving two mechanisms:

(1) “Vegetation–climate negative feedback regulation mechanism”: The increase of vegetation cover X7 (vegetation cover) and X6 (normalized vegetation factor) can alleviate the negative impact of X3 (dryness index), and enhance the buffering capacity of the system against drought and extreme temperatures. For example, the Sanjiang Plain wetland restoration project reduced the risk of extreme drought events by increasing evapotranspiration to regulate the local climate. This is similar to the findings of Marisa scholars and others [17].
(2) “Human-land system coupling” [15]: The adaptive management approach of “investing economic resources in ecological restoration projects” is highlighted by the interaction effects of the irrigation index (X9) and GDP per capita (X12). This is consistent with the findings of Yu Chunzhe [68] and Ding Zhaoyi [69], which imply that the combined effects of various natural circumstances and human activities are responsible for the heterogeneity of the regional and temporal distribution of ecological vulnerability.

It is important to note that in 2022, the dryness index (X3)‘s single-factor explanatory power fell precipitously, but its interaction effect remained strong. This is indicative of the shift in the driving mode of climate factors, whereby the explanatory power of pure climate indicators decreased while their coupling effect with human factors increased. This relates to Northeast China’s agricultural adaptation strategy over the last ten years: the socioeconomic system more indirectly expresses the effects of climate factors, while human intervention, such as water-saving irrigation (X9) and planting structure adjustment (X6), partially eliminates the direct effects of drought.

Furthermore, in contrast to earlier research [57], this work focuses more intently on the Northeast agricultural ecology. The chosen indicators include agriculture data in addition to ecological indicators [22]. This paper presents the agricultural and ecological vulnerability zoning of the Northeast region at the prefecture-level scale, focuses on analyzing the driving factors of vulnerability in the agricultural ecosystem of the Northeast, and examines the spatiotemporal characteristics of vulnerability at various periods. In light of this, this research suggests the following actions to stably lessen the agricultural ecosystem’s susceptibility in Northeast China: (1) Implement a differentiated ecological compensation mechanism: High-vulnerability areas should receive high-standard compensation focused on curbing degradation and ecological restoration, with strong measures taken to prevent further deterioration of ecosystems; medium-vulnerability areas should receive moderate compensation focused on transforming production methods, such as subsidies for conservation tillage; low-vulnerability areas should focus on market-based compensation that incentivizes continuous improvement, with an emphasis on cultivating green production market mechanisms. (2) Prioritize the strengthening of surface vegetation regulation capacity, focusing on enhancing ecological resilience by optimizing ecological compensation policies, promoting protective farming techniques (e.g., straw mulching), constructing farmland protection forest belts, and implementing differentiated arable land fertility protection subsidies for low-vegetation-covered areas, such as the Liao-Zhong-South urban agglomeration. (3) Optimize the direction of socio-economic drive: promote moderate-scale operation through land transfer to reduce the pressure of urbanization on marginal arable land; identify hotspots of urban expansion relying on nighttime lighting data, and delineate agro-ecological red lines. (4) Focus on emerging risk factors: In response to the continuous rising trend of the irrigation index factor, it is necessary to implement a smart irrigation system to prevent and control the risk of secondary salinization triggered by over-exploitation of water resources. (5) Improve interaction management: Establish a synergistic monitoring network of vegetation cover, climate drought and economic inputs, and utilize their interactive effects to design adaptive farming systems.

However, this paper still has problems that need to be improved. First, 13 evaluation indicators were selected to assess the vulnerability of agroecosystems in Northeast China. Although a large number of references were made to the literature in selecting evaluation indicators and calculating weights, the factors leading to vulnerability are relatively numerous and complex, so the constructed evaluation indicator system may have limitations. Secondly, this paper uses linear interpolation to handle a small amount of missing data (such as GDP), which may not accurately capture complex dynamics such as nonlinearity, leading to bias in the interpolation point estimates and affecting the accuracy of the research results. Finally, while the optimal parameter-based geodetector solves the discretization problem, it is unable to capture the intricate and nonlinear relationships between several ecological driving forces. In conclusion, more comprehensive indicators should be taken into consideration in the future to deepen the scale and multi-dimensional study of the spatial and temporal characteristics and driving factors of vulnerability. This will help to provide a reference and a basis for decision-making for the realization of sustainable development and ecological protection of agro-ecosystems in Northeast China.

## 5. Conclusions

(1) In terms of temporal evolution, the overall vulnerability of agricultural ecosystems in Northeast China has shown a fluctuating downward trend. The proportion of different vulnerability levels has fluctuated significantly, with moderately vulnerable areas continuously transitioning toward mild vulnerability, indicating a positive trend.
(2) In terms of spatial evolution, the vulnerability of the agricultural ecosystem in the northeastern region shows a distribution pattern of “with vulnerability concentrated in the southwest and declining toward the northeast” gradually decreasing from south to north, with an overall trend of gradual improvement.
(3) In terms of driving forces, the spatial pattern is mainly determined by the terrain, with surface vegetation playing a core regulatory role by buffering climate pressures and enhancing ecological functions. At the same time, agricultural production conditions serve as a key human intervention to enhance resilience. This relies on the deep interaction between surface vegetation and human activity factors, which, through feedback between ecological and economic systems, and synergy between vegetation and irrigation, jointly drive the system’s resilience pattern.

## Supporting information

S1 File (ZIP)
