# Supplementary figures and images for: Agroecosystem vulnerability and driving factors in Northeast China
Source: PLoS One. 2026 Feb 12;21(2):e0339870. doi: 10.1371/journal.pone.0339870 (PMC12900346; doi:10.1371/journal.pone.0339870)

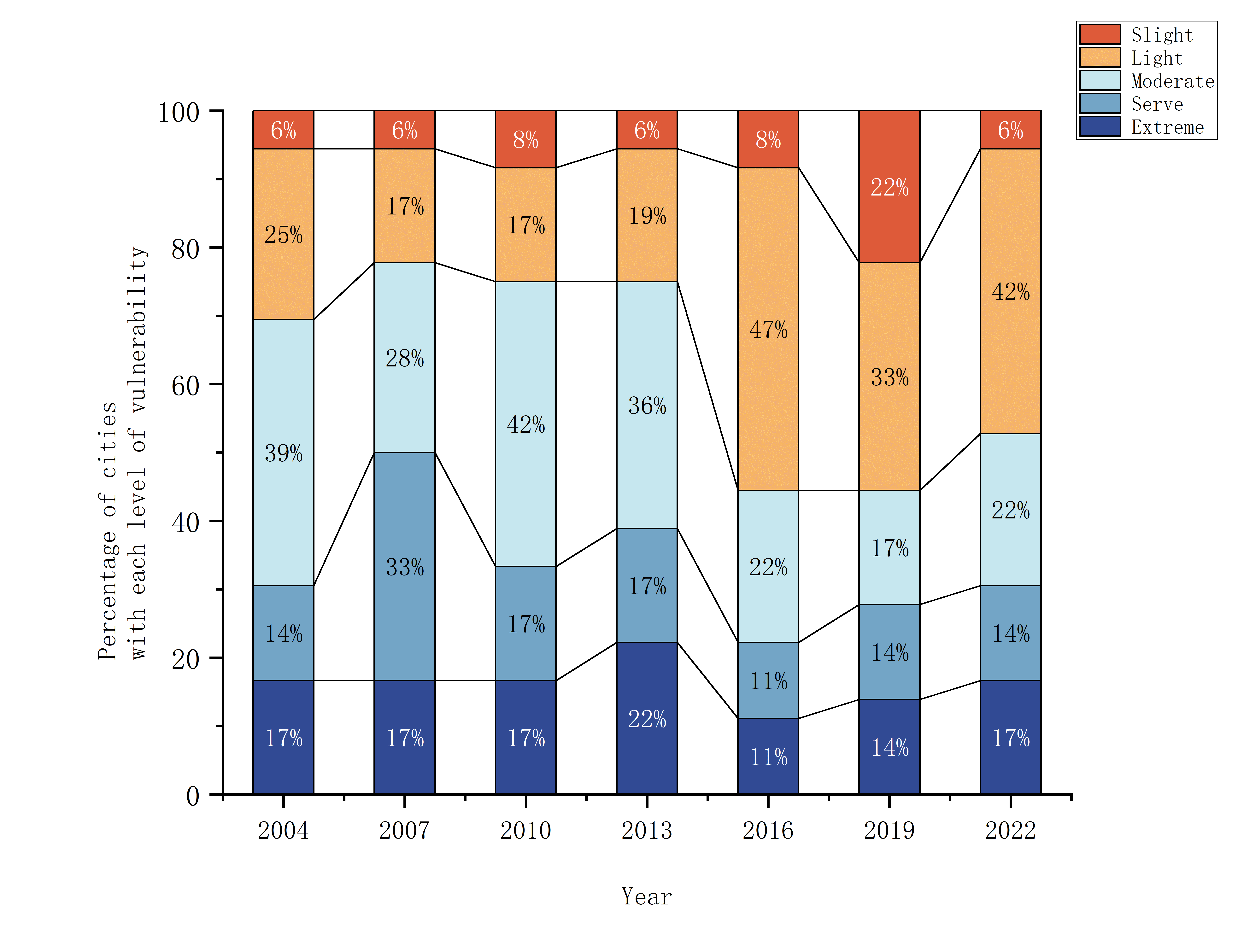

Supplement: S1 File — (ZIP) [file pone.0339870.s001.zip › Figure.tif]

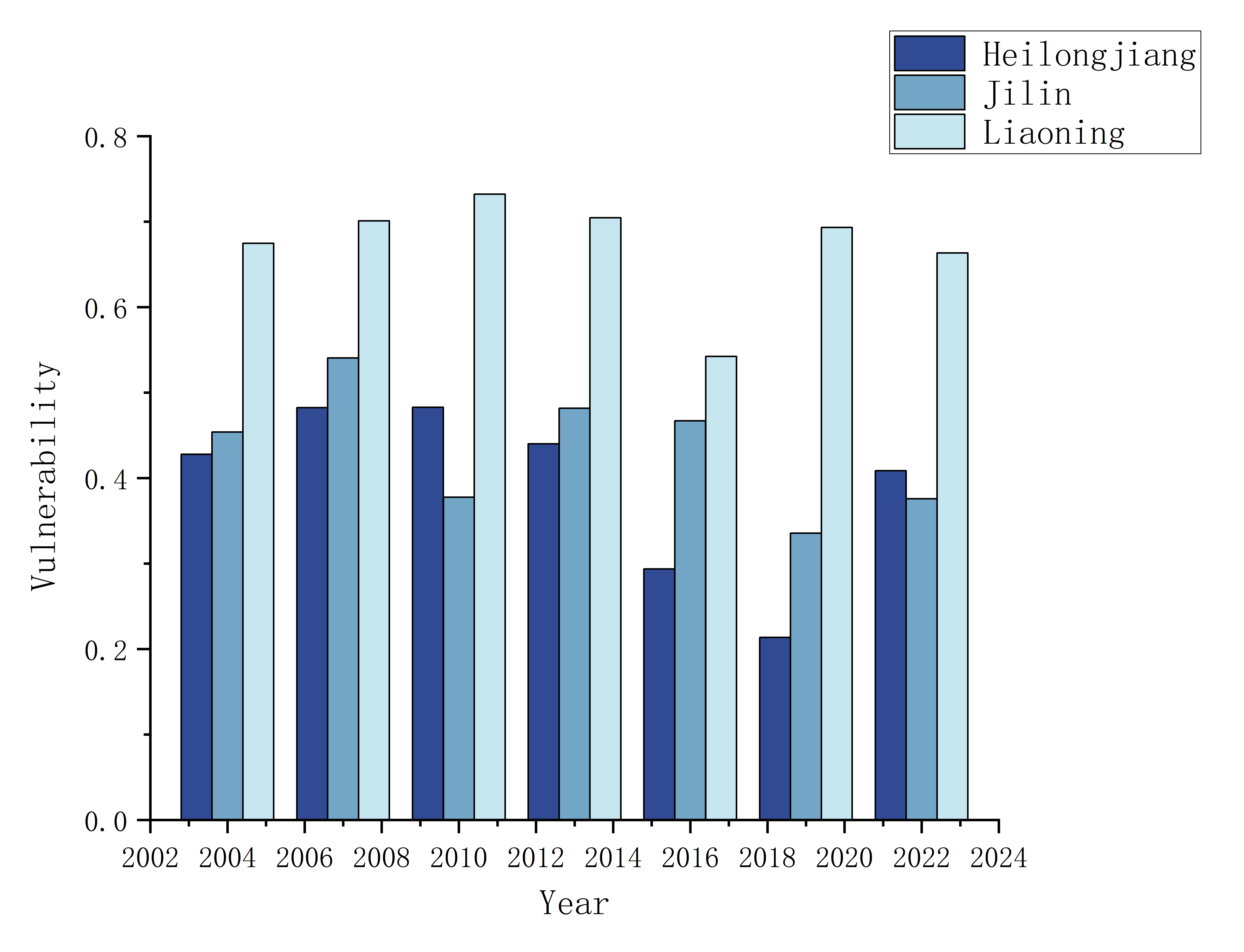

Supplement: S1 File — (ZIP) [file pone.0339870.s001.zip › Figure2.tif]

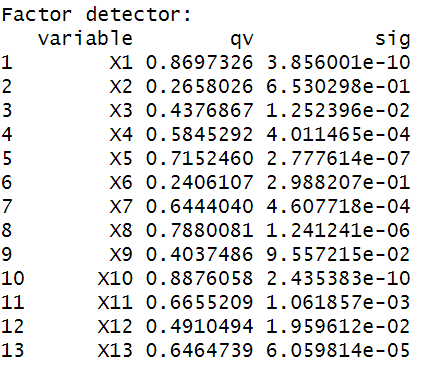

Supplement: S1 File — (ZIP) [file pone.0339870.s001.zip › Geodetector/2004 output.png]

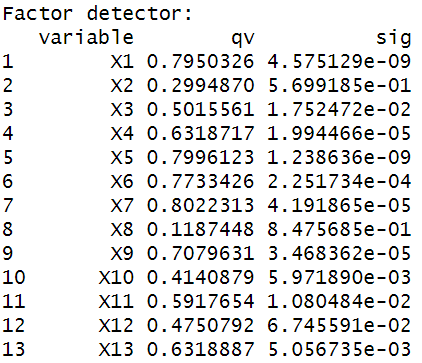

Supplement: S1 File — (ZIP) [file pone.0339870.s001.zip › Geodetector/2013 output.png]

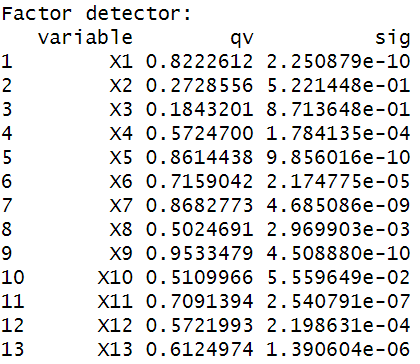

Supplement: S1 File — (ZIP) [file pone.0339870.s001.zip › Geodetector/2022 output.png]
